# Central Thalamic-Medial Prefrontal Control of Adaptive Responding in the Rat: Many Players in the Chamber

**DOI:** 10.3389/fnbeh.2021.642204

**Published:** 2021-04-08

**Authors:** Robert G. Mair, Miranda J. Francoeur, Brett M. Gibson

**Affiliations:** ^1^Department of Psychology, University of New Hampshire, Durham, NH, United States; ^2^Neural Engineering and Translation Lab, University of California, San Diego, San Diego, CA, United States

**Keywords:** central thalamus, prefrontal cortex, reward guided, mediodorsal nucleus of thalamus, intralaminar nuclei of the thalamus, anterior cingulate (ACC), conditional discrimination, midline thalamic nuclei

## Abstract

The medial prefrontal cortex (mPFC) has robust afferent and efferent connections with multiple nuclei clustered in the central thalamus. These nuclei are elements in large-scale networks linking mPFC with the hippocampus, basal ganglia, amygdala, other cortical areas, and visceral and arousal systems in the brainstem that give rise to adaptive goal-directed behavior. Lesions of the mediodorsal nucleus (MD), the main source of thalamic input to middle layers of PFC, have limited effects on delayed conditional discriminations, like DMTP and DNMTP, that depend on mPFC. Recent evidence suggests that MD sustains and amplifies neuronal responses in mPFC that represent salient task-related information and is important for detecting and encoding contingencies between actions and their consequences. Lesions of rostral intralaminar (rIL) and ventromedial (VM) nuclei produce delay-independent impairments of egocentric DMTP and DNMTP that resemble effects of mPFC lesions on response speed and accuracy: results consistent with projections of rIL to striatum and VM to motor cortices. The ventral midline and anterior thalamic nuclei affect allocentric spatial cognition and memory consistent with their connections to mPFC and hippocampus. The dorsal midline nuclei spare DMTP and DNMTP. They have been implicated in behavioral-state control and response to salient stimuli in associative learning. mPFC functions are served during DNMTP by discrete populations of neurons with responses related to motor preparation, movements, lever press responses, reinforcement anticipation, reinforcement delivery, and memory delay. Population analyses show that different responses are timed so that they effectively tile the temporal interval from when DNMTP trials are initiated until the end. Event-related responses of MD neurons during DNMTP are predominantly related to movement and reinforcement, information important for DNMTP choice. These responses closely mirror the activity of mPFC neurons with similar responses. Pharmacological inactivation of MD and adjacent rIL affects the expression of diverse action- and outcome-related responses of mPFC neurons. Lesions of MD before training are associated with a shift away from movement-related responses in mPFC important for DNMTP choice. These results suggest that MD has short-term effects on the expression of event-related activity in mPFC and long-term effects that tune mPFC neurons to respond to task-specific information.

## Introduction

To survive in a dynamic environment organisms must be able to adapt efficiently to changes in conditions, responding in ways that optimize favorable consequences. Behavioral ecologists have demonstrated that foraging animals select among food patches of different quality in a way that maximizes food intake while reducing energy costs (Stephens and Krebs, 1986). Animals often have to simultaneously evaluate other negative and positive factors such as predation risk and mating opportunities (Nonacs, 2001), making seemingly simple decisions more complicated. Rats utilize complex strategies to optimize food acquisition, weighing information about the size and location of food items, exposure, deprivation, circadian time, risk of predation, and possible theft by conspecifics (Whishaw and Dringenberg, 1991; Whishaw et al., 1992). Wood mice in the wild exploit information about food access, experience with the food source, and predation risk in making foraging decisions (Navarro-Castilla et al., 2018; Hernández et al., 2019). Many species learn to exploit heterospecific alarm calls to evade potential predators and thus increase their foraging efficiency (Magrath et al., 2015). Eastern gray squirrels shift energy towards vigilance and away from foraging following exposure to red-tailed hawk calls and use subsequent bird chatter as a cue to safety (Lilly et al., 2019). Evolution has equipped organisms with neural mechanisms that allow them to choose a course of action likely to produce favorable consequences based on current goals, past experiences, updated information about action-outcome contingencies, and sensory evidence. This requires the ability to integrate allocentric information about the external world with egocentric information about the organization and execution of actions, internal state conditions, the anticipation of likely outcomes, and assessment of the actual consequences of behavior.

**Figure 1:**
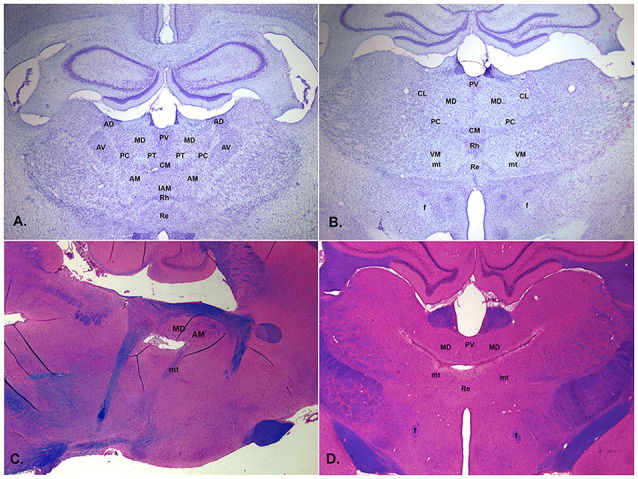
Low magnification photomicrographs of central thalamus showing normal appearance of coronal sections stained with thionin approximately 2.3 mm **(A)** and 2.8 mm **(B)** posterior to bregma and thalamic lesions from the post-thiamine deficiency (PTD) model of the Wernicke-Korsakoff syndrome (WKS) stained with luxol blue, hematoxylin, and eosin in the sagittal section about 0.9 mm off midline (**C**, tissue from Mair et al., 1988) and in the coronal section about 3.2 mm posterior to bregma (**D**, tissue from Mair et al., 1991). PTD treatment produces bilaterally symmetric lesions centered on the mediodorsal (MD) and intralaminar nuclei that tend to spare the anterior nuclei. It is associated with behavioral impairments consistent with the performance of WKS patients on comparative neuropsychological tasks (Mair, 1994). Labeled structures include the MD, centrolateral (CL), paracentral (PC), central medial (CM), reuniens (Re), rhomboid (Rh), ventromedial (VM), anterodorsal (AD), anteromedial (AM), anteroventral (AV), interanteromedial (IAM), and paratenial (PT) nuclei and the fornix (f) and mammillothalamic tract (mt).

Medial prefrontal cortex (mPFC) plays a critical role in adaptive goal-directed behavior (Miller and Cohen, 2001; Dalley et al., 2004; Chudasama, 2011; Kesner and Churchwell, 2011; Balleine, 2019). mPFC has robust afferent and efferent connections with multiple thalamic nuclei that are clustered in the central thalamus and are hence referred to as central thalamic nuclei (Figure 1). Central thalamic nuclei are elements in large-scale networks connecting mPFC with the hippocampus, basal ganglia, amygdala, other areas of the neocortex, and visceral and arousal systems in the brainstem that give rise to adaptive goal-directed behavior. They include the paraventricular (PV), paratenial (PT), reuniens (Re), and rhomboid (Rh) midline nuclei; the central medial (CM), central lateral (CL), and paracentral (PC) rostral intralaminar nuclei; the anterior medial (AM) and interanteromedial (IAM) anterior nuclei; and the mediodorsal (MD) and ventromedial (VM) nuclei (Groenewegen, 1988; Sesack et al., 1989; Berendse and Groenewegen, 1991; Ray and Price, 1992; Vertes, 2002, 2004; Hoover and Vertes, 2007). Although early studies focused on MD, the main source of thalamic input to middle layers of mPFC, it is now clear that MD has limited effects on mPFC function and that other central thalamic nuclei contribute importantly to the effects of mPFC on goal-directed behavior (Dalley et al., 2004; Balleine and O’Doherty, 2010; Chudasama, 2011; Euston et al., 2012; Mitchell et al., 2014; Mair et al., 2015; Marton et al., 2018; Parnaudeau et al., 2018; O’Mara and Aggleton, 2019; Wolff and Vann, 2019; McGinty and Otis, 2020).

Vertes et al. (2015) classified a group of nuclei along the thalamic midline as limbic based on prominent afferent and efferent connections with limbic-related structures and evidence that they serve limbic-related functions, including affective behaviors, reward-guided responding, response-related working memory, and behavioral flexibility. These nuclei include PV, PT, Re, Rh, and CM nuclei and the medial division of MD. PC, CL, and more lateral divisions of MD are reciprocally connected to more dorsal motor-related areas in anterior cingulate and agranular medial areas of mPFC (Groenewegen, 1988; Berendse and Groenewegen, 1991; Ray and Price, 1992; Vertes, 2002; Hoover and Vertes, 2007). Recent evidence indicates that thalamocortical neurons in MD are strongly excited by the driver and modulatory input from PFC and indirectly influence reciprocally-connected neurons in PFC by enhancing cortical connectivity and regulating neuronal activity (Barbas et al., 1991; Xiao et al., 2009; Schmitt et al., 2017; Collins et al., 2018). The projections of the rostral intralaminar nuclei have thalamocortical and thalamostriatal projections to areas that are interconnected by corticostriatal projections: connections that appear organized to control interactions between mPFC and the basal ganglia and thus selection of goals, actions, and associative stimuli (Berendse and Groenewegen, 1990; Groenewegen and Berendse, 1994; Grillner et al., 2005; Mannella et al., 2016). The intralaminar and midline nuclei receive prominent subcortical inputs from periaqueductal gray, parabrachial nuclei, superior colliculus, hypothalamus, and brainstem nuclei (Krout and Loewy, 2000a, b; Krout et al., 2001, 2002; Bayer et al., 2002). These provide signals related to visceral, nociceptive, orienting, and arousal functions consistent with a role for these nuclei in behavioral state control of mPFC function (Kinomura et al., 1996; Schiff and Purpura, 2002; Mair and Hembrook, 2008).

The ventromedial nucleus (VM) has dense reciprocal connections with the agranular medial cortex and adjacent motor and cingulate areas. Afferent inputs to VM include branches of axons that also innervate MD and GABAergic projections from the basal ganglia. Thalamocortical neurons in VM have dense widespread projections to layer 1 in agranular medial and adjacent motor and cingulate cortices and less dense projections in parietal and occipital cortices that appear organized to control integrative motor responses (Vertes, 2002; Hoover and Vertes, 2007; Kuramoto et al., 2017; Collins et al., 2018; Sierveritz et al., 2019). The anterior thalamic nuclei receive inputs from the subicular complex of the hippocampal formation and are reciprocally connected to the retrosplenial cortex, an important hub for spatial cognition. The IAM and AM nuclei are also reciprocally connected to anterior cingulate and prelimbic areas of mPFC (Vertes, 2002; Hoover and Vertes, 2007). Lesions of the anterior thalamic nuclei affect allocentric spatial learning tasks (Aggleton and Nelson, 2015; O’Mara and Aggleton, 2019).

Anatomical analyses indicate that rodent mPFC is homologous to primate anterior cingulate and premotor cortices and lacks an area homologous to primate dlPFC (Preuss, 1995; Uylings et al., 2003; Vogt et al., 2013; Schaeffer et al., 2020). Here we focus on interactions between mPFC and thalamus in rodents to avoid the complexity of factoring in the influence of primate dlPFC on thalamocortical interactions. In this article, we review the two convergent approaches that have been used to elucidate the role of thalamo-prefrontal pathways in generating adaptive responses. The first is to lesion or manipulate the activity of these pathways and study the effects on behavioral measures of adaptive responding. Here we use behavioral measures to examine the influence of different thalamic nuclei on functions supported by mPFC. The second is to record the activity of mPFC and central thalamic neurons in awake, behaving animals to understand what information is represented and how cortical and thalamic neurons interact during adaptive goal-directed behavior. Here, we emphasize recordings comparing mPFC with MD, given the lack of data to support comparisons with other central thalamic nuclei. We focus on spatial delayed conditional discriminations: tasks that have received considerable attention in both behavioral and electrophysiological recording studies. These tasks incorporate important features of adaptive goal-directed responding: flexible reward-guided choice, where different responses are reinforced on different trials; conditional discrimination, where a preceding stimulus indicates which response alternative will be reinforced; working memory, where information must be represented briefly in memory; spatial navigation, where behavioral events are distributed topographically; and motor planning, where organisms must organize and execute a series of actions to obtain reinforcement. Lesion studies in rats have provided evidence that pathways connecting mPFC with the striatum, pallidum, and thalamus are critical for spatial delayed conditional discrimination (Dunnett, 1990; Reading and Dunnett, 1991; Kesner et al., 1996; Mair et al., 1998, 2002; Floresco et al., 1999; Burk and Mair, 2001a; Porter et al., 2001; Bailey and Mair, 2005; Zhang et al., 2005; Sloan et al., 2006).

## Behavioral Studies of mPFC Function

Early studies of delayed response deficits in monkeys (Jacobsen, 1936) led investigators to focus on the role of the prefrontal cortex in working memory: the ability to hold information online and guide behavior with these internal representations (Goldman-Rakic and Selemon, 1997; Fuster, 2001). Subsequent studies identified the principal sulcus of monkey dlPFC as a critical site for visuospatial working memory (Goldman and Rosvold, 1970; Goldman et al., 1971) and ventral lateral PFC for non-spatial working memory (Goldman-Rakic, 1996; Meyer et al., 2011). Early lesion studies in the rat associated mPFC with impairments of spatial reversal, delayed response, and delayed alternation and orbitofrontal PFC with increased perseveration and responding during extinction: findings consistent with effects of dlPFC and orbitofrontal cortex lesions in non-human primates (Divac, 1971; Kolb et al., 1974; Larsen and Divac, 1978). These results led to the view that portions of rodent mPFC are homologous to primate dlPFC. Anatomical studies have challenged that view by demonstrating homology between rodent mPFC and primate anterior cingulate and premotor cortices, but not dlPFC, based on relative location, cytoarchitecture, receptor binding studies, and anatomical and functional connectivity (Preuss, 1995; Vogt et al., 2013; Schaeffer et al., 2020).

### Effects of mPFC Lesions on Spatial Delayed Conditional Discrimination

**Figure 2:**
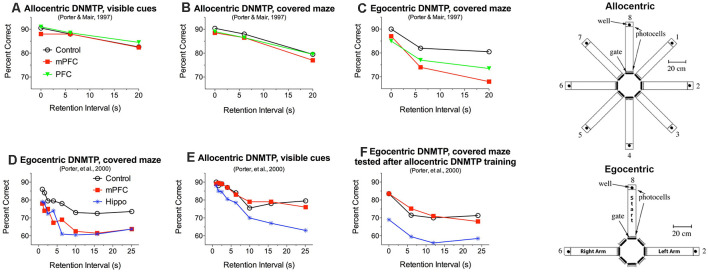
Effects of medial prefrontal (mPFC), complete prefrontal (PFC), and hippocampal (Hippo) lesions on allocentric and egocentric versions of 2-choice DNMTP trained in eight arm radial mazes. Samples and choices in egocentric DNMTP were between the same two arms on every trial, located to the left and right of the holding arm. Arms for allocentric DNMTP were selected at random on every trial from the eight alternatives so that their configurations were unpredictable. For egocentric DNMTP external cues were minimized by covering mazes and training in the dark, while allocentric DNMTP was trained in uncovered mazes with lights on and many visible external cues. mPFC and PFC lesions impaired egocentric **(C,D)** and spared allocentric DNMTP **(A,B,E)** while hippocampal lesions impaired both tasks **(D,E)**. Rats that received extensive allocentric training (30 presurgical and 20 postsurgical sessions) were protected when subsequently switched to egocentric DNMTP **(F)**. Data are replotted from studies cited.

Working memory is important for adaptive responding: allowing organisms to hold and manipulate information online while monitoring fluctuations in the environment (Miller et al., 2018; Cavanaugh et al., 2020). Early lesion studies indicated that mPFC lesions in the rat have a selective effect on working memory, impairing delayed conditional discriminations based on response-related egocentric information while sparing maze tasks that require an allocentric solution (Becker et al., 1980; Kolb et al., 1983; Kesner et al., 1996; de Bruin et al., 2001). Porter and Mair (1997) tested this distinction by comparing the effects of PFC lesions on a series of tasks trained in automated 8 arm radial mazes, beginning with an allocentric 8 arm task trained in a lighted room with many visible cues and changing in stages to end with two choice egocentric DNMTP (Figure 2). Neither mPFC nor complete PFC lesions affected the allocentric tasks, including two choice DNMTP where three arms were selected at random for each trial from the eight arms of the maze (i.e., no predictable configuration between the arms) both with and without visible external cues (Figures 2A,B). Both mPFC and complete PFC lesions produced stable impairment only at the last stage of training where the same three arms were used on every trial in a T-configuration such that choice responses were defined by the egocentric direction of turning (left vs. right) from the stem of the T and the room was darkened and mazes covered to eliminate external allocentric visual cues (Figure 2C). Porter et al. (2000) confirmed these findings by comparing the effects of mPFC and hippocampal lesions on the allocentric and egocentric versions of 2-choice radial maze DNMTP. Both hippocampal and mPFC lesions produced delay-independent impairment for rats trained pre-surgically and tested post-surgically with the egocentric version (Figure 2D) while hippocampal lesions produced delay-dependent impairment and mPFC lesions spared the allocentric version (Figure 2E). To test possible explanations for deficits, tasks were switched after initial post-surgical testing. Interestingly, rats trained initially with the allocentric version of DNMTP were protected from effects of mPFC, but not hippocampal, lesions when switched to the egocentric version (Figures 2D,F).

These findings have several important implications. First, since egocentric and allocentric versions of radial maze DNMTP used the same deprivation procedures and water reinforcement it is unlikely that the egocentric impairment is related to reinforcement mechanisms or to the ability to utilize reinforcement-related information flexibly to select different response alternatives on different trials. Second, the ability of mPFC lesioned rats to perform the egocentric version following extensive allocentric training (Figure 2F) indicates that the deficits observed for egocentric DNMTP (Figures 2C,D) cannot be ascribed to the repetition of the same response alternatives on every trial. This rules out interference effects or difficulty of temporal discrimination produced by frequent repetition of response alternatives. One possibility consistent with these results is that rats were biased by pre-and post-surgical allocentric training to rely on allocentric cues or an internalized spatial map of the maze when subsequently switched to egocentric DNMTP (see Porter et al., 2000 for a discussion of this possibility).

Third, the spared ability of rats with mPFC or complete PFC lesions to perform allocentric DNMTP shows that the effects of mPFC lesions on egocentric DNMTP deficits cannot be ascribed to a generalized impairment of working memory. mPFC lesions in these studies involved agranular medial, anterior cingulate, and prelimbic cortices while the complete PFC lesions additionally damaged agranular insular and ventral orbital areas (cortical areas based on Öngür and Price, 2000; Heidbreder and Groenewegen, 2003). These results are consistent with reports that similar mPFC lesions impair egocentric while sparing allocentric spatial memory in other tasks (Kolb et al., 1983; Harrison and Mair, 1996; Kesner et al., 1996; de Bruin et al., 2001) and, further, spare other measures of working memory, including olfactory continuous non-matching-to-sample (Koger and Mair, 1994) and visual object memory (Kesner et al., 1996; Ennaceur et al., 1997). Ragozzino et al. (1998, 2002), report that more ventral mPFC lesions involving IL and MO areas affect working memory for allocentric spatial and visual object information. These findings are consistent with the view that different regions of PFC support different domains of working memory (Goldman-Rakic, 2005; Kesner and Churchwell, 2011). More recent evidence has shown that different modalities of working memory are mediated by other areas of the association cortex (Miller et al., 2018; Xu, 2018; Buchsbaum and D’Esposito, 2019; Cavanaugh et al., 2020) indicating that working memory may represent a property of cortex that extends beyond PFC.

The delay-independent effects of mPFC lesions on DMTP and DNMTP (Figures 2, 4H) are consistent with evidence that PFC lesions affect the ability to use conditional or other learned rules to select between choice alternatives independent of demands on working memory (Petrides, 1985; Winocur and Eskes, 1998; Sharpe and Killcross, 2015; Germann and Petrides, 2020). Stevens and Mair (1998) tested this idea with an auditory match-to-position (AMTP) task in which the discriminative stimulus was turned off at different times before (mnemonic) or after (non-mnemonic) the choice response. While all groups performed better for non-mnemonic conditions where stimuli were present at the time of choice, mPFC and central thalamic lesions produced comparable deficits for mnemonic and non-mnemonic choice. These results demonstrate an impairment in a trial-to-trial selection based on a learned conditional rule where the discriminative stimulus is present at the time of choice. They do not rule out a role for working memory in monitoring actions and outcomes or utilizing learned information about the conditional rule in nonmnemonic AMTP trials. Analyses of DMTP (Mair et al., 1998) and DNMTP (Harrison and Mair, 1996; Porter et al., 2000) in operant chambers that allow precise measurement of response time (RT) data have revealed significant increases in choice RT for mPFC lesions (Figure 5D). Other analyses in these studies showed that matching for RT did not affect deficits in response accuracy. Thus mPFC lesions appear to have two distinct effects on egocentric spatial delayed conditional discriminations: delay-independent impairment of response accuracy and slower RTs for choice responses.

**Figure 3:**
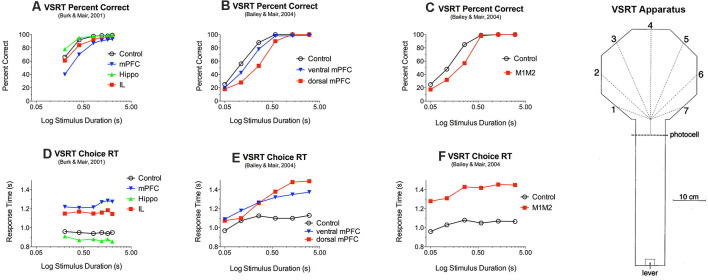
Effects of medial prefrontal (mPFC) and motor (M1M2) cortex and rostral intralaminar (IL) thalamic lesions on reaction time (RT) and accuracy of responding to luminance cues in the visuospatial reaction time (VSRT) task. VSRT trials were initiated by a lever press at the end of the arm. A brief luminance cue was then triggered to indicate the S+ response port when the rat crossed the photocell just before entering the octagonal hub. Water reinforcement was delivered in the S+ port if the rat entered it first within a 3.0 s limited hold. PFC and M1M2 lesions had duration-dependent effects on response accuracy **(A,B,C)** and increased RT for **(D,E,F)**. IL thalamic lesions increased RT **(D)** but did not have a significant effect on choice accuracy **(A)**. Data are replotted from studies cited.

**Figure 4:**
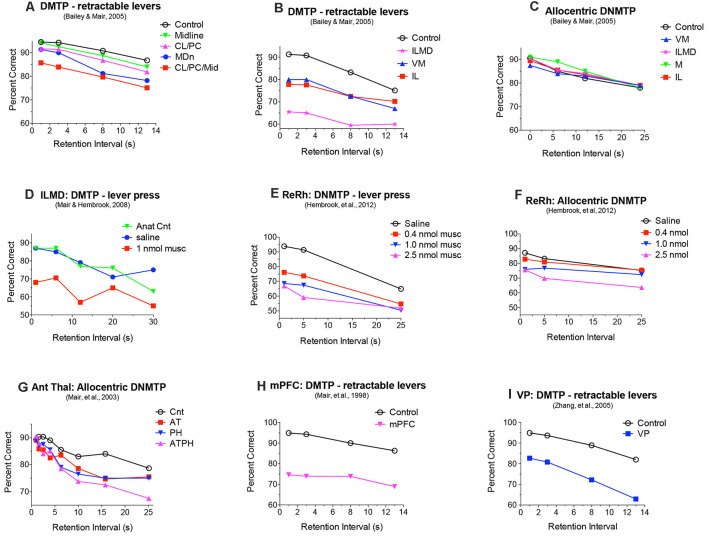
Effects of central thalamic lesions on delayed matching (DMTP) and non-matching (DNMTP) to position accuracy. Lesions of the mediodorsal nucleus (MD) produced delay-dependent **(A)** and lesions of rostral intralaminar (IL), ventromedial (VM), and IL and MD combined (ILMD) produced delay-independent impairment **(B)** of egocentric DMTP trained with retractable levers, an impairment also observed with reversible inactivation of IL and MD **(D)**. IL, ILMD, and VM lesions did not have significant effects on allocentric radial maze DNMTP **(C)**. Reversible inactivation of reuniens (Re) and rhomboid (Rh) nuclei in the ventral midline thalamus produced delay-independent impairment of DNMTP trained with retractable levers at all muscimol doses tested **(E)** and allocentric radial maze DNMTP only at the highest dose tested **(F)**. Anterior thalamic (AT) lesions produced delay-dependent impairment of allocentric radial maze DNMTP comparable to the effects of parahippocampal cortex (PH) lesions **(G)**. Combined AT and PH lesions produced a larger deficit, comparable to the effects of hippocampal lesions Figure 2E on this task. Lesions of the medial prefrontal cortex (mPFC) and ventral pallidum (VP) produce delay-independent impairment of DMTP **(H,I)** comparable to the effects of IL, ILMD, and VM lesions **(B)**. Data are replotted from studies cited.

**Figure 5:**
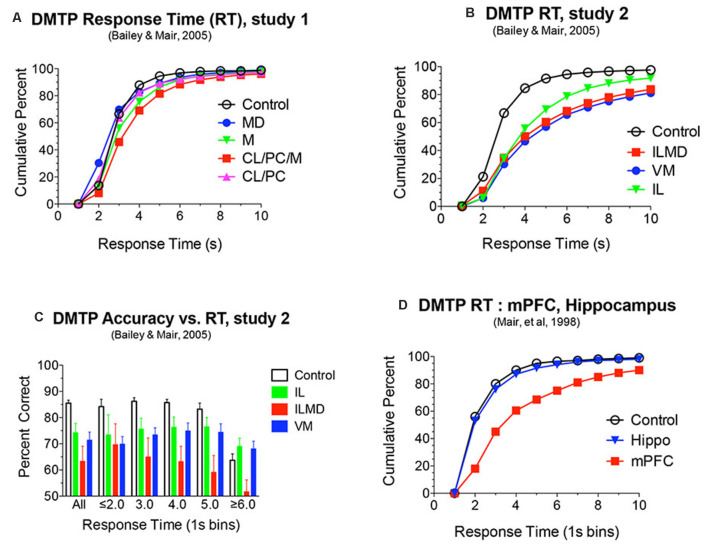
**(A,B,D)** Response time (RT) analyses of DMTP choice plotted as cumulative functions. RT was measured from the lever press marking the end of the memory delay to the choice press (see Figure 6). RT was increased significantly by rostral intralaminar (IL), ventromedial (VM), and large lesions damaging IL and the mediodorsal (MD) nuclei (ILMD; **B**). These same lesions produced delay independent impairments of DMTP (Figure 4B). To test whether the increase in RT could account for the decrease in DMTP accuracy, separate analyses were conducted for responses divided into discrete RT bins. Restricting responses to 1 s time bins did not affect significant deficits produced by each of the lesions for RT bins with sufficient numbers of responses to support these analyses **(C)**. mPFC, but not hippocampal, lesions were associated with a similar increase in DMTP RT **(D)** and accuracy Figure 4H. Data are replotted from studies cited.

### Effects of mPFC Lesions on Instrumental Behavior

mPFC lesions that produce DMTP (Mair et al., 1998) or DNMTP (Harrison and Mair, 1996; Porter et al., 2000) impairments have been found to spare discrimination learning when tested subsequently for serial reversal learning where choices involved the same levers, in the same apparatus, with the same reinforcement used for the DMTP or DNMTP tasks. This suggests that mPFC lesions that affect delayed conditional discrimination spare rule-based discrimination learning where the same response alternative is associated with reinforcement on every trial. Other evidence suggests that mPFC lesions can affect rule-based responding when stimuli are difficult to discriminate (Bussey et al., 1997) or under conditions that make it difficult to detect and attend to information relevant to action selection (Birrell and Brown, 2000; Dalley et al., 2004; Chudasama, 2011; Fisher et al., 2020; Bubb et al., 2021). For instance, mPFC lesions increase RT and decrease accuracy for responding to brief luminance cues in the 5-choice serial reaction time task (Muir et al., 1996; Chudasama et al., 2003). Interestingly, more posterior (Muir et al., 1996) or ventral (Chudasama et al., 2003) mPFC lesions were found to increase premature responding in this task, indicative of impaired inhibitory control.

Similar results have been observed for a visual-spatial reaction time (VSRT) designed around the octagonal hub used for radial DNMTP tasks (Figure 3). In VSRT trials, rats entered the hub from the observation arm (see Figure 3), triggering a brief luminance cue that indicated the correct response port where a nose poke was reinforced with water. In DNMTP rats entered a similar hub from the holding arm where they were presented with open gates for two arms. They received water reinforcement for responding to the arm not reinforced on the preceding sample trial (Figure 2; see above). mPFC lesions increased RT and impaired accuracy of VSRT and produced egocentric radial maze DNMTP impairment consistent with earlier results shown in Figure 2 (Bailey and Mair, 2004). Lesions of adjacent M1 and M2 motor cortex also affected VSRT accuracy and RT but spared egocentric DNMTP. Thus, mPFC lesions affected the trial-to-trial selection of spatially defined response alternatives following learned conditional rules in VSRT and egocentric DNMTP, while M1M2 lesions affected sensory-, but not a memory-guided choice. The effects of M1M2 lesions on VSRT are consistent with evidence linking motor and premotor cortices with control of intentional movements guided by external stimuli (Brown et al., 1991; Muir et al., 1996; Georgopoulos and Carpenter, 2015).

It has been argued that mPFC is important for evaluating actions and outcomes along multiple dimensions (Skvortsova et al., 2014). The anterior cingulate area of mPFC has been implicated in discriminating the utility of different reward options in effort-based decision-making (Walton et al., 2006; Hart et al., 2020). Consistent with this view the prelimbic area of mPFC has been implicated in the ability to detect action-outcome contingencies (Balleine and O’Doherty, 2010; Balleine, 2019). Balleine and Dickinson (1998) first showed that prelimbic lesions make instrumental responding insensitive to outcome devaluation. Subsequent work found that these effects only occur with lesions made before initial training (Ostlund and Balleine, 2005; Hart and Balleine, 2016). Thus, the prelimbic cortex appears to be critical for encoding action-outcome associations but is not the location where they are stored (Balleine, 2019). More recently, Alcaraz et al. (2018) used chemogenetic methods to dissect the contributions of thalamocortical and corticothalamic projections to dorsal mPFC on encoding action-outcome encoding. They report that thalamocortical projections are important for both outcome- and contingency-devaluation, while corticothalamic projections are important for outcome-, but not contingency-devaluation.

Fuster (2001) argued that PFC is organized to remember, plan, and execute actions: integrating perceptions and actions in time to support goal-directed behavior. Consistent with this mPFC lesions affect recency discriminations used to assess temporal order memory in the rat (Kesner and Churchwell, 2011). Other evidence indicates that prefrontal and premotor cortices interact with striatum to support action sequence learning in humans and non-human primates (Kennerley et al., 2004; Poldrack et al., 2005; Di Russo et al., 2017). Bailey and Mair (2006, 2007) showed comparable effects of frontal cortical and striatal lesions in rats trained to perform a series of nose poke responses in an action sequence learning task. For unlesioned controls repetition learning increases RT to initiate learned sequences, reflecting motor planning, and decreased RT for subsequent responses in the sequence, reflecting the benefits of habitual learning. Lesions involving agranular medial and anterior cingulate areas of mPFC increased RT to initiate learned sequences, suggesting a role for these areas in motor preparation, while sparing the decrease in RT for later nose pokes in learned sequences, providing evidence of spared habitual repetition learning. Parallel effects were observed for lesions in related areas of the striatum (Bailey and Mair, 2006).

Adaptive goal-directed behavior depends on multiple functions supported by mPFC. There is strong evidence for the role of mPFC in working memory, although it seems unlikely that this extends across all domains of information or that it is sensitive to the length of the memory delay. Beyond remembering stimulus information, working memory is important for monitoring actions and outcomes across time (see Dalley et al., 2004), holding stimulus information online to control attention, and maintaining goal-related information during motor planning. mPFC is important for flexible responding where trial-to-trial response selection is based on a learned conditional rule. mPFC has also been implicated in several attentive processes that allow animals to respond efficiently to task-relevant information. A case could also be made for a fundamental role of mPFC in detecting and encoding relationships between actions and their consequences and thus the capacity for adaptive goal-directed responding. Such impairment could potentially account for the profound effects of mPFC lesions on conditional responding, particularly for tasks that require flexible selection of different responses on different trials. Finally, mPFC is also thought to play an important role in organizing and executing temporal sequences of actions.

## Behavioral Studies of Central Thalamic Function

mPFC interacts with multiple central thalamic nuclei to control neural networks that give rise to adaptive goal-directed behavior (Mitchell et al., 2014; Mair et al., 2015; Halassa and Sherman, 2019; Fresno et al., 2019). To what extent do individual nuclei contribute to mPFC control of intentional responding? Early studies focused on MD, in part because of prominent connections with PFC and its implication in early studies of amnesia (Victor et al., 1971; Isseroff et al., 1982; von Cramon et al., 1985; Zola-Morgan and Squire, 1985). Several lines of research broadened this focus to include contributions of other central thalamic nuclei: anatomical evidence that multiple central thalamic nuclei have afferent and efferent connections with PFC and PFC-related pathways (Groenewegen, 1988; Sesack et al., 1989; Berendse and Groenewegen, 1991; Ray and Price, 1992; Vertes, 2004; Hoover and Vertes, 2007); clinical studies associating thalamic amnesia with damage to other parts of central thalamus (Carlesimo et al., 2011; Van der Werf et al., 2003); findings from the post-thiamine deficiency (PTD) model of the Wernicke-Korsakoff syndrome that intralaminar lesions produce cognitive impairment in this model (Figures 1C,D; Mair, 1994; Mair et al., 2015); and evidence that the hippocampal-anterior thalamic axis plays a critical role in episodic memory (Aggleton and Brown, 2006). A complete review of this literature is beyond the scope of this article. Here we will focus on response-related measures of learning and memory that depend on mPFC in the rat.

### Effects of Intralaminar, Mediodorsal, and Ventromedial Nuclei on Spatial Delayed Conditional Discrimination

Large central thalamic lesions involving intralaminar nuclei and adjacent areas of MD produce delay-independent impairment comparable to mPFC lesions, affecting both the speed and accuracy of DMTP and DNMTP choice (Mair and Lacourse, 1992; Burk and Mair, 1998). Similar deficits were observed for the PTD model (Mair et al., 1991; Robinson and Mair, 1992) where MD and intralaminar nuclei are consistent sites of thalamic pathology (Figure 1). Reversible inactivation of these nuclei with microinjected drugs can produce comparable impairment of accuracy without affecting response speed (Figure 4D; Porter et al., 2001; Mair and Hembrook, 2008). To control for the effects of response accuracy, and thus the frequency of reinforcement, on deficits produced by thalamic lesions a staircase method was used to define the memory delay producing 75% accuracy for PTD (Robinson and Mair, 1992) and radiofrequency thalamic lesions (Mair and Lacourse, 1992). This method was successful in matching lesion and control groups for response accuracy (and thus reinforcement density) while demonstrating significant impairment in the length of retention interval producing 75% accuracy for both PTD (6.1 vs. 14.6 s for controls) and radiofrequency lesions (7.4 vs. 17.8 s).

Discrete lesions targeting specific nuclei have more specific effects. Bailey and Mair (2005) compared the effects on DMTP of lesions targeting MD, intralaminar, midline, and ventromedial (VM) nuclei that were carefully positioned to avoid damaging anterior thalamic nuclei. Lesions restricted to lateral intralaminar (CL, PC) or dorsal midline nuclei did not significantly affect performance alone, but combined in the larger CL/PC/Mid lesion produced a delay independent impairment (Figure 4A) that did not affect response time (RT; Figure 5A). Lesions restricted to MD resulted in delay-dependent deficits that did not affect RT. Burk and Mair (1998) found a non-significant trend towards impairment and no effect on RT for MD lesions with a similar DMTP task in which sample response requirements were manipulated. Other studies examining the effects of MD on spatial memory tasks have produced mixed results for rats and monkeys (Mitchell and Chakraborty, 2013). Young et al. (1996) found a delay-dependent impairment for MD lesions in a fine-grained analysis of temporal decay in an operant DNMTP task where rats were trained to stability at a series of delays and rate of decay inferred from an asymptotic performance at each delay. By contrast, Hunt and Aggleton (1998) found significant effects of MD lesions on errors to criterion learning DMTP trained in a T-maze but not for temporal decay of this task once learned. Clearly, MD lesions do not produce DMTP or DNMTP deficits comparable to the more substantial, delay-independent effects of mPFC lesions which affect accuracy and RT for these tasks (Figures 2, 4, 5).

Bailey and Mair (2005) found that complete intralaminar lesions involving CL, PC, and CM produced delay independent deficits for DMTP accuracy (Figure 4B) and RT (Figure 5B) comparable to effects of mPFC lesions. VM lesions also produced delay independent deficits with a more substantial increase in RT. Large ILMD lesions, involving midline, MD, and intralaminar nuclei caused delay independent impairment about twice as severe as intralaminar (or VM) lesions (Figure 4B) and increased RT comparable to VM lesions (Figure 5B). To test whether deficits in DMTP or DNMTP accuracy are secondary to effects of thalamic lesions on response speed, separate analyses of response accuracy were conducted with restricted RT windows (Mair and Lacourse, 1992; Burk and Mair, 1998, 1999; Bailey and Mair, 2005). The results of these analyses have consistently shown effects of thalamic lesions on DMTP and DNMTP accuracy persist with RT restrictions except for long RTs where group differences are limited by floor effects (Figure 5C). Intralaminar lesions have broad effects on adaptive responding that can affect functions spared by mPFC lesions. These include olfactory continuous non-matching to sample (Koger and Mair, 1994; Zhang et al., 1998) and serial reversal learning (Mair et al., 1991; Harrison and Mair, 1996; Burk and Mair, 1998).

Bolkan et al. (2017) used optogenetic methods to demonstrate a delay-dependent effect of MD inhibition in mice for T-maze DNMTP. Interestingly they found evidence for a directional interaction where thalamocortical projections of MD support sustained firing in mPFC during the memory delay and cortico-thalamic projections the subsequent choice response. Other recent studies have shown that persistent cortical activity depends on thalamocortical loops involving MD and mPFC for attentional control (Schmitt et al., 2017) and VM for preparatory activity in the motor cortex (Guo et al., 2017). Collins et al. (2018) used optogenetics to dissect cortico-thalamocortical networks involving MD and VM. They report that MD and VM are excited by reciprocally-connected neurons providing layer 5 “driver” and layer 6 “modulatory” afferents from mPFC, that appear organized to activate and maintain persistent firing in thalamocortical neurons. Collins et al. also found that thalamocortical projections of MD strongly activate layer 2/3 cortico-cortical neurons while VM provides subthreshold excitation across layers in mPFC. The predominant thalamocortical and corticothalamic connections between VM and dorsal agranular medial areas of mPFC (Vertes, 2002; Hoover and Vertes, 2007) seem consistent with the implication of VM supporting preparatory responses in the motor cortex (Guo et al., 2017). Taken together these results suggest that cortico-thalamocortical circuits support the temporary maintenance of information in mPFC important for adaptive responding that extends well beyond the traditional view of working memory as a temporary buffer for sensory or episodic information.

### Effects of Midline and Anterior Nuclei on Spatial Delayed Conditional Discrimination

Intralaminar lesions that extend into anterior areas of the thalamus produce delay independent impairment of allocentric radial maze DNMTP (Mair et al., 1998), a task spared by intralaminar lesions that do not affect anterior thalamus (Bailey and Mair, 2005; see above). The anterior thalamic nuclei are important nodes in hippocampal-related pathways that support allocentric spatial learning and memory (Aggleton and Nelson, 2015; O’Mara and Aggleton, 2019). Multiple reports indicate that anterior thalamic lesions affect allocentric DNMTP and other measures of spatial memory spared by mPFC, intralaminar, and MD lesions (Warburton et al., 1997; Mair et al., 2003; Wolff et al., 2008). Mitchell and Dalrymple-Alford (2006) compared effects of anterior thalamic lesions with lateral thalamic lesions involving intralaminar and MD and found evidence of a double-dissociation consistent with these findings: anterior thalamic lesions affected the post-surgical acquisition of an allocentric spatial memory task trained in a radial maze and spared performance of a pre-surgically trained egocentric memory task, while lateral lesions of MD and the intralaminar nuclei had opposite effects. Alcaraz et al. (2016) reported an analogous double dissociation in which lesions damaging MD and intralaminar nuclei affected a spatial outcome-devaluation task while sparing an allocentric spatial memory task, while anterior nucleus lesions had the opposite effects. The severe effects of anterior thalamic lesions on spatial function have been attributed to different functions mediated by individual anterior thalamic nuclei as well as “covert effects” of anterior thalamic lesions on distributed hippocampal-related networks (Aggleton and Nelson, 2015). While AM and IAM are reciprocally connected to mPFC, these connections are not critical for egocentric function spared by anterior thalamic lesions and insufficient to disrupt allocentric function spared by mPFC lesions.

The reuniens (Re) and rhomboid (Rh) nuclei in the ventral midline thalamus are important sources of thalamic input to the hippocampus and mPFC that appear organized to modulate mPFC—hippocampal interactions (Vertes et al., 2006). Early evidence showed that these nuclei are important for spatial memory tasks that depend on both mPFC and hippocampus. Lesions damaging Re and Rh affect radial maze measures of spatial memory while sparing visually-guided choice in VSRT and action sequence learning (Hembrook and Mair, 2011). Localized inactivation of Re and Rh with low doses of muscimol (0.4 or 1.0 nmol) affects operant DNMTP, a task sensitive to the effects of both mPFC and hippocampal lesions, while sparing allocentric radial maze DNMTP, a task sensitive to the effects of hippocampal but not mPFC lesions (Hembrook et al., 2012). Subsequent studies have confirmed an important role for Re and Rh in aspects of spatial memory and cognitive control that requires the coordinated activity of the hippocampus and mPFC (Dolleman-van der Weel et al., 2019; Mathiasen et al., 2020). Both the anterior thalamic and ventral midline Re and Rh are organized to support interactions between mPFC and hippocampus. Anterior thalamic nuclei, with strong retrosplenial, cingulate, and mammillary body connections, appear specialized to support allocentric hippocampal function. Re and Rh, with their extensive connections with the hippocampus and mPFC, appear specialized to support interactions between them (Dolleman-van der Weel et al., 2019; Mathiasen et al., 2020).

### Effects of Thalamic Lesions on Instrumental Behavior

Intralaminar lesions increase RT without affecting the accuracy of conditional responses to brief luminance cues in the VSRT task, while mPFC lesions increase RT and decrease accuracy (see above), and hippocampal lesions had no significant effect on RT or accuracy (Burk and Mair, 2001b). Other reports indicate that both MD (Chudasama and Muir, 2001) and Re (Prasad et al., 2013) lesions increase premature responding without affecting responses to luminance cues in the 5 choice task. This suggests a role for MD and Re on inhibitory control, but not sensory attention. The effects of intralaminar lesions on VSRT RT are consistent with evidence that intralaminar, but not MD, lesions affect choice RT for DMTP and DNMTP tasks (Figures 5A,B; see above). The effect of MD lesions on premature responding is consistent with the effects of lesions damaging the prelimbic area of mPFC.

Lesion studies have also demonstrated parallel effects of MD and prelimbic cortex lesions on action-outcome learning. Thus, MD lesions were found to abolish the effects of outcome devaluation (Corbit and Balleine, 2003). Like prelimbic lesions, MD lesions affect outcome devaluation only when made before initial training (Ostlund and Balleine, 2008). Thus, both prelimbic and MD appear to be essential for the acquisition, but not the expression, of goal-directed behavior. The importance of connections between the prelimbic cortex and MD for action-outcome learning was confirmed by a crossed lesion study, where lesions damaging MD in one hemisphere, PL in the other, and contralateral projections of MD in the corpus callosum affected comparable to bilateral MD or prelimbic lesions on outcome devaluation (Bradfield et al., 2013). Recently, Alcaraz et al. (2018) used chemogenetic methods to provide evidence that thalamocortical projections from lateral MD to dorsal mPFC affect both outcomes- and contingency-devaluation, while corticothalamic pathways between these areas affect the outcome- but not contingency-devaluation.

Dorsal midline lesions damaging the PV and PT nuclei have not been associated with significant effects on egocentric DNMTP (Mair and Lacourse, 1992) or DMTP tasks (Bailey and Mair, 2005; Figures 4A, 5A). They have extensive limbic-related connections with systems that are important for instrumental behavior, including inputs from visceral-, arousal-, and emotion-related areas in the brainstem, hypothalamus, and limbic forebrain and projections to prelimbic and infralimbic areas of mPFC, agranular insular and entorhinal cortices, subiculum, nucleus accumbens, and striatum, and the extended amygdala (Krout and Loewy, 2000a, b; Krout et al., 2001, 2002; Bayer et al., 2002; Vertes and Hoover, 2008; Kirouac, 2015). PV and PT thus appear organized to integrate information related to the behavioral state to influence brain systems that support adaptive goal-directed behavior. Functional analyses have focused more on PV than PT. These have provided evidence that PV affects reward-seeking behavior (McGinty and Otis, 2020), control of wakefulness (Ren et al., 2018), the salience of stimuli related to reward, aversion, novelty, and surprise in associative learning (Zhu et al., 2018), and conditioned and unconditioned emotional behavior (Barson et al., 2020). Lesion studies have shown that PV affects the attribution of incentive salience to reward cues in sign tracking (Haight et al., 2015).

Adaptive goal-directed responding requires organisms to plan and execute actions based on current and remembered information about the external environment, internal state conditions, and action-outcome contingencies. Central thalamic nuclei link mPFC with multiple neural networks that support these functions. No individual nucleus has proven critical for mPFC function in general. Nevertheless, lesions of specific nuclei can account for some effects of mPFC lesions, showing the importance of thalamocortical and cortico-thalamic pathways in these functions. For instance, intralaminar and VM lesions produce delay-independent impairments comparable to mPFC, affecting RT and accuracy of egocentric DMTP and DNMTP. Lesion studies have also shown that some nuclei that are reciprocally connected with mPFC can affect functions spared by mPFC lesions. For instance, anterior thalamic lesions affect measures of allocentric DNMTP spared by mPFC lesions. This suggests that mPFC can influence functions of thalamic circuits without being a critical node in the circuit, presumably through top-down control. To elucidate functional interactions between the thalamus and cortex it is important to move beyond behavioral analyses and examine the information represented by neurons in these pathways during adaptive goal-directed behavior and how this is influenced by cortico-thalamic and thalamocortical projections.

## What Information Is Represented by mPFC Neurons During Adaptive Goal-Directed Behavior?

Lesion studies have revealed important roles for mPFC and adjacent motor cortices in reward-guided learning and decision-making and provided evidence that different subregions of mPFC support specific aspects of reward-guided responding. Lesions of more dorsal areas affect memory for motor responses, response selection, and reward-guided choice while more ventral lesions of PL and IL affect allocentric and visual memory and supervisory attentional control (Dalley et al., 2004; Chudasama, 2011; Kesner and Churchwell, 2011). To what extent do mPFC neurons represent information related to functions affected by mPFC lesions? Are there abrupt transitions in the response properties of mPFC neurons that correspond with behavioral functions ascribed to dorsal and ventral subregions?

**Figure 6:**
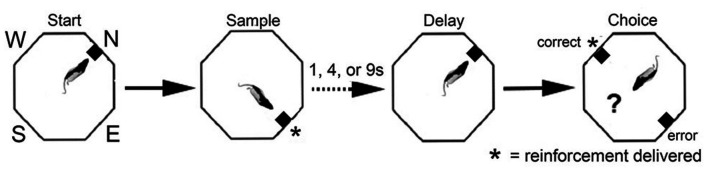
Schematic drawing of dynamic DNMTP (dDNMTP) task. Training occurred in open octagonal chambers, equipped with levers on four walls, 90° apart, with a drinking spout above each lever to deliver reinforcement. Trials began with a randomly selected lever (N, E, S, or W) extending for the start response. The sample and correct choice levers were 90° to the left or right of the start lever, which also served as the delay lever (extending at the end of the memory delay to initiate the choice response). The length of the retention interval and the direction of the sample lever (L vs. R) were randomly selected for each trial. Reinforcement was given following the sample press and when the non-matching to sample lever was pressed during the choice phase. See text for details.

Single unit recordings in awake, behaving rats have revealed mPFC neurons with responses related to movement, actions, preparation to respond, anticipation and delivery of rewards, errors, working memory delay, and spatial location during different tasks (Jung et al., 1998; Pratt and Mizumori, 2001; Chang et al., 2002; Baeg et al., 2003; Hok et al., 2005; Euston and McNaughton, 2006; Cowen and McNaughton, 2007; Totah et al., 2009, 2013; de Saint Blanquat et al., 2010; Euston et al., 2012; Horst and Laubach, 2012; Hyman et al., 2012, 2013; Powell and Redish, 2014; Insel and Barnes, 2015). We developed the dynamic DNMTP (dDNMTP) task to examine neuronal responses related to each of these functions in a single task incorporating features known to be sensitive to mPFC lesions (Figure 6). dDNMTP is trained in open octagonal arenas with retractable levers and spouts for water reinforcement on four walls 90° apart (N, E, S, W). Trials consist of a series of four lever presses. The sample phase begins with a base lever (randomly selected for each trial) extending for the start. This retracts when pressed and the sample lever extends (90° to the left or right randomly selected). This retracts when pressed and water reinforcement is delivered from the spout immediately above. After a memory delay (randomly selected for each trial) the base lever extends again for the delay response. This retracts when pressed and the levers 90° to the left and right extend for the choice response. When the lever not extended for the sample is pressed reinforcement is delivered (*) and the levers retracted. The dDNMTP choice is egocentrically defined like DMTP and DNMTP tasks affected by mPFC lesions. The open arena provides sufficient space to characterize the movement-related neuronal activity and visible cues to examine allocentric spatial coding. By starting trials at randomly selected locations, responses related to the spatial location can be distinguished from behavioral events which shift in location for trials beginning at different base levers.

**Figure 7:**
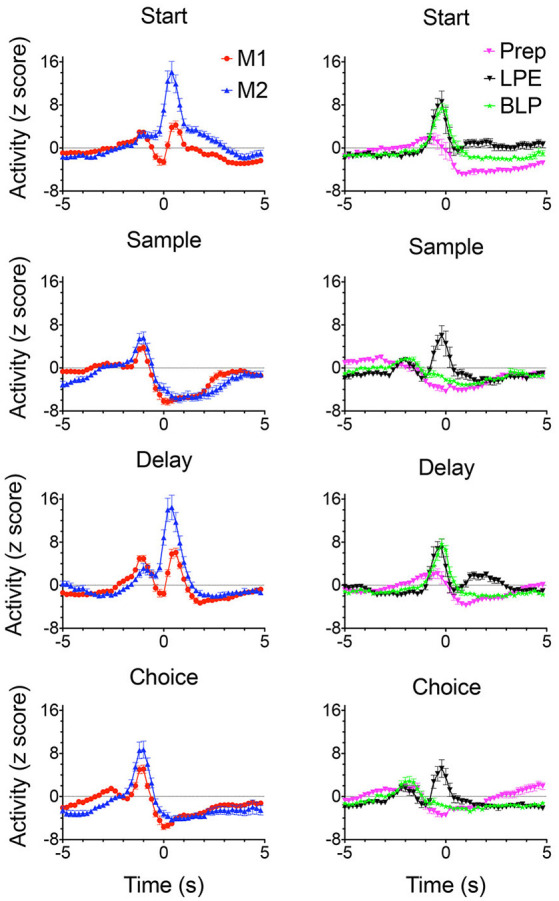
Normalized population PETHs based on all examples observed of action-related responses. Movement 1 (M1) fired during movements toward each lever in the sequence (*N* = 97). Movement 2 (M2) fired during movements toward the sample and choice levers and were directionally specific (*N* = 32). Lever press excitation (LPE) fired during each of the 4 lever presses in the sequence (*N* = 28). Base lever press (BLP) fired during start and delay lever presses (*N* = 30). Preparatory (Prep) exhibited activity that ramped up to a peak just before the start and (to a lesser extent) the delayed responses (*N* = 44). Activity is aligned with each of the lever presses and plotted for 5 s before until 5 s after the aligned event. Error bars represent the standard error of the mean. Data are replotted from Francoeur and Mair (2018).

**Figure 8:**
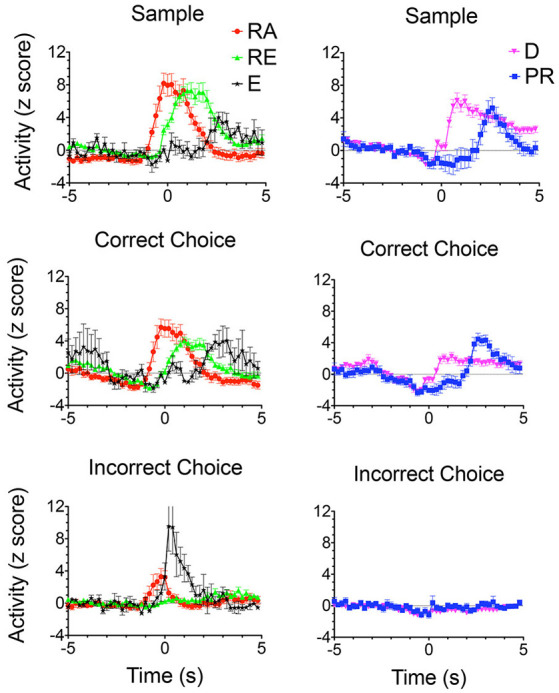
Normalized population PETHs based on all examples observed of outcome-related responses. Each of these responses differentiated reinforced sample and correct choice responses from unreinforced incorrect choices. Reinforcement anticipation (RA) responses began 0.6–0.8 s before sample and choice responses and lasted until 1.6 s after reinforcement began or 0.2 s after errors when reinforcement was not delivered (*N* = 50). Reinforcement excitation (RE) began when reinforcement was delivered and lasted an average of 2.7 s (*N* = 63). Error (E) responses lasted for an average of 1.6 s after incorrect responses when expected reinforcement was not delivered (*N* = 4). Delay-related responses exhibited increased activity lasting across the memory delay, on average from 0.4 to 5.0 s after sample reinforcement and 0.2–1.6 s following correct choice reinforcement (*N* = 58). Some delay-related had spatially-restricted firing patterns (Figures 9A,B,F) and thus potentially carried information about the location of sample reinforcement across the delay interval sufficient to support a correct DNMTP choice. Post-reinforcement responses lasted on average from 2.3 to 3.6 s after reinforcement began, a time when rats tended to disengage after consuming reinforcement from the drinking spout (*N* = 16). Activity is aligned with each of the lever presses and plotted for 5 s before and after the aligned event. Error bars represent the standard error of the mean. Data are replotted from Francoeur and Mair (2018).

Of 1,335 isolated neurons recorded with moveable tetrode arrays, 458 (34.3%) exhibited criterion event-related activity of which 445 (33.3%) exhibited temporal patterns of activity related to actions or outcomes that were characterized as normalized population peri-event time histograms (PETH; Figures 7, 8). These included preparation to respond, movement between levers, lever-press responses, reinforcement anticipation, reinforcement delivery, errors, and memory delays. Action-related responses (Figure 7) included 129 that fired during periods of movement, 58 that fired during lever press responses, and 44 that fired during preparation before the start response. Movement-related responses included neurons that fired during all periods of movement between levers (M1; *n* = 97) and others that were directionally specific and fired during movements from the base to the sample and from the base to the choice levers (M2; *n* = 32). Lever press-related responses included neurons firing during all four lever presses (LPE; *n* = 28) and others that fired only during base lever presses (BLP; *n* = 30).

Outcome related responses (*N* = 191) included reinforcement anticipation (RA; *n* = 50) that fired beginning 0.7 s before predictable times of reward and persisted for an average of 2.7 s throughout reward delivery; reinforcement excitation (RE, *n* = 63) that fired within 0.2 s after reward delivery and remained elevated for an average of 3.0 s; error responses (E; *n* = 4) that fired within 0.2 s of when the expected reward was not delivered; delay (D) responses (*n* = 58) that started firing within 0.4 s of when sample rewards were delivered and continued until the delay lever press; and post-reinforcement (PR) responses (*n* = 16) that began after reward delivery ended when rats disengaged from drinking spouts where rewards were delivered.

**Figure 9:**
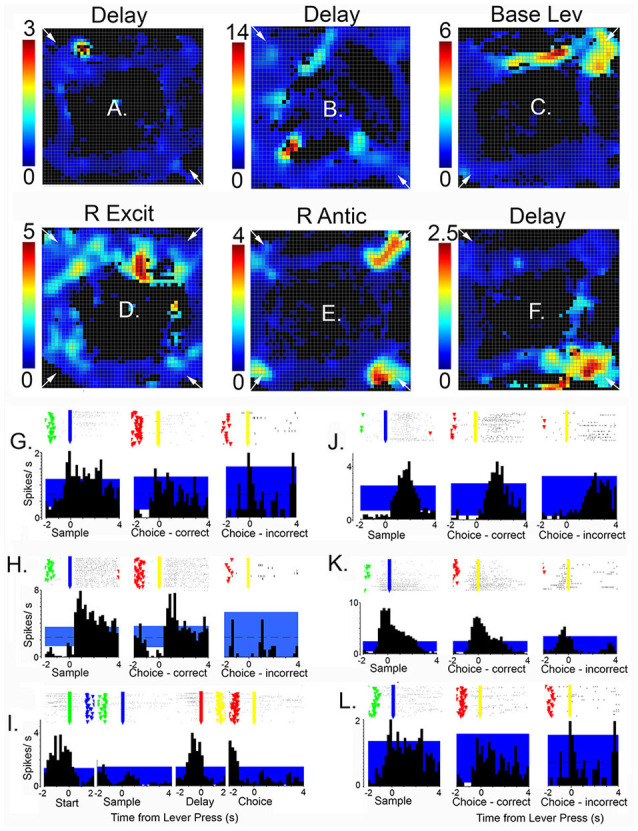
Heat maps, raster plots, and peri event time histograms (PETHs) of neurons exhibiting spatially-restricted event-related responses. Results are shown for six neurons, three with delay-related responses **(A,G,B,H,F,L)**, along with examples of base lever press **(C,I)**, reinforcement excitation **(D,J)**, and reinforcement anticipation **(E,K)**. Heat maps are oriented with levers/drinking spouts in each corner. Calibration bars to the left indicate activity in spikes/s. Rasters show activity in trial 1 at the bottom and trial 60 at the top with event markers for lever presses (green, blue, red, and yellow for the start, sample, delay, and choice lever presses, respectively). Time scales and aligned events are matched for PETHs and overlying raster plots. The blue band marks the 99% confidence interval for PETHs. This figure is reproduced from Onos et al. (2016).

Spatial mapping of neuronal activity during dDNMTP revealed areas of activation consistent with event-related analyses, thus neurons firing during lever presses or reinforcement have higher activity in locations of response levers, and reward spouts and movement-related responses are associated with elevated activity on pathways between levers. Some neurons fire in all possible locations where associated events occur while others are spatially-restricted, firing in a subset of possible locations. Figure 9 (from Onos et al., 2016) shows examples of spatially-restricted responses. Spatial heat maps and event-related rasters and PETHs are shown for three neurons with delay related responses **(A,G; B,H**; and **F,L)** along with single examples for base lever press **(C,I)**, reinforcement excitation **(D,J)**, and reinforcement anticipation **(E,J)**. Although the data were insufficient for vector or decoding analyses (Georgopoulos and Carpenter, 2015; Yin et al., 2018) these results are consistent with a population code representing information about actions and their location in allocentric space.

**Figure 10:**
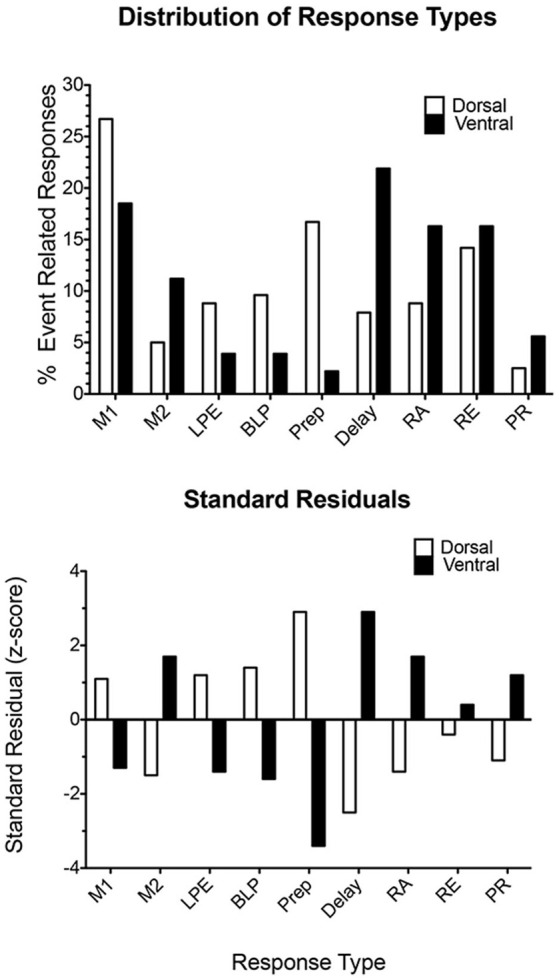
Distribution of response types observed in dorsal and ventral areas of mPFC. The upper plot compares the percent of responses in each compartment exhibiting each of the most common response types observed during dDNMTP. The lower plot shows standard residuals indicating the contributions of each response type to the significant chi-square comparing the distribution of different response types in dorsal and ventral mPFC. Abbreviations are the same as for Figure 11. Figure is reproduced from Francoeur and Mair (2018).

Histological analyses show substantial overlap between the distributions of all response types in mPFC. Statistical analyses revealed biases between dorsal and ventral mPFC, with dorsal areas having more neurons with motor-related responses, including movement between all levers (M1), lever presses (both LPE and BLP), and preparatory responses (Figure 10). Ventral areas had relatively high concentrations of neurons firing during movement towards rewards (M2), delay periods following reinforcement, reinforcement anticipation (RA), and post-reinforcement (PR). There was a relatively even distribution of neurons firing during reinforcement delivery (RE) consisting of about 15% of event-related responses in dorsal and ventral compartments (Francoeur and Mair, 2018). Thus, while there are differences in the broad distributions of different response types between dorsal and ventral mPFC, there was no evidence of an abrupt transition in the types of information represented in different regions of mPFC.

Each of the response types observed in rodent mPFC during dDNMTP represents task-specific aspects of goal-directed behavior that are consistent with mPFC functions identified by behavioral analyses of lesion effects. PFC relies on working memory to temporarily maintain information not available to the senses to support adaptive goal-directed responding. This is thought to be represented by persistent neuronal firing during delay intervals (Fuster and Alexander, 1971; Fuster, 2001; Goldman-Rakic, 2005) although evidence has been presented that working memory may also be implemented by sequential neuronal activation (Rajan et al., 2016). Working memory for cognitive information is associated with delay-related activity in primate dlPFC (Fuster and Alexander, 1971; Fuster, 2001; Goldman-Rakic, 2005). Enel et al. (2020) have recently presented evidence that delay-related activity in monkey anterior cingulate cortex, the likely homolog of rodent mPFC (Preuss, 1995; Vogt et al., 2013; Schaeffer et al., 2020), represents information about reward value. This is consistent with the finding of delay period activity related to reinforcement delivery in mPFC during the dDNMTP task (Figure 8). At least three other response types represent information not immediately available to the senses presumably held in working memory. These include preparatory responses before the start response, reinforcement anticipation before sample and choice responses, and post-reinforcement responses.

The preponderance of neurons with responses related to actions and outcomes is consistent with evidence implicating mPFC in action-outcome contingency: clearly, mPFC neurons represent information required for this function. Similarly, the concentration of neurons with preparatory responses in dorsal mPFC for dDNMTP (Figure 7) and other tasks (Jung et al., 1998; Chang et al., 2002; Totah et al., 2009, 2013) is in keeping with evidence that lesions here selectively affect the initiation of learned action sequences (Bailey and Mair, 2007). The importance of mPFC for organizing temporal sequences of behavior is supported by normalized population PETHs (Figures 7, 8) that reveal a cascade of tightly coupled neuronal responses that effectively tile the temporal interval between initial preparation to when rats disengage from reinforcement following choice responses. Finally, recording studies have consistently shown large populations of neurons tuned to respond to task-relevant information. Here 445/1,335 isolated neurons exhibited one of ten discrete response types related specifically to the arbitrary actions and outcomes of dDNMTP (Figures 7, 8). Similar numbers are reported for the proportion of mPFC neurons exhibiting task-specific event-related activity for different behavioral tasks (Jung et al., 1998; Pratt and Mizumori, 2001; Chang et al., 2002; Baeg et al., 2003; Hok et al., 2005; Euston and McNaughton, 2006; Cowen and McNaughton, 2007; Totah et al., 2009, 2013; de Saint Blanquat et al., 2010; Euston et al., 2012; Horst and Laubach, 2012; Hyman et al., 2012, 2013; Powell and Redish, 2014; Insel and Barnes, 2015). These tuning properties of mPFC neurons seem consistent with evidence that mPFC lesions affect the ability of rats to detect and discriminate information relevant to adaptive action selection (Birrell and Brown, 2000; Dalley et al., 2004; Chudasama, 2011; Fisher et al., 2020; Bubb et al., 2021).

Electrophysiological analyses of neuronal activity in awake, behaving animals support the homology of rodent mPFC with primate anterior cingulate and premotor cortices. Premotor neurons in monkeys have been shown to encode movement-related information in extrinsic coordinates related to actions rather than muscle-like activity (Kakei et al., 2001, 2003), with imprecise coding of directional or spatial information in single neurons that presumably rely on population coding to achieve precision (Georgopoulos and Carpenter, 2015; Yin et al., 2018). Motor responses are preceded by preparatory activity related to motor planning (Shenoy et al., 2013; Murakami and Mainen, 2015). Other reports have described neuronal responses that precede expected reinforcement or mark the delivery or absence of expected reinforcement in monkey motor, premotor, and anterior cingulate cortices (Roesch and Olson, 2003; Amiez et al., 2006; Matsumoto et al., 2007; Marsh et al., 2015) as well as human medial PFC (Domenech et al., 2020). Recently, Enel et al. (2020) reported that delay-related activity in monkey anterior cingulate represents information about the expected value of action outcomes. This homology is also consistent with recent results from resting-state fMRI analyses that rodent mPFC has stronger connections with motor areas of cortex than the more broadly distributed connections of primate mPFC: an organization in the rat more closely related to premotor than dlPFC areas of primates (Schaeffer et al., 2020).

## How Do Central Thalamic Nuclei Influence Event-Related Responses of mPFC Neurons?

Cortical projections excite the thalamus through driver and modulatory projections from layers 5 and 6 of mPFC. Thalamic projections activate excitatory cortico-cortical neurons and inhibitory interneurons to enhance cortical connectivity and thus regulate the activity of mPFC neurons (Cruikshank et al., 2012; Rovó et al., 2012; Bolkan et al., 2017; Schmitt et al., 2017; Collins et al., 2018; Huo et al., 2020; Lee et al., 2020). To what extent do central thalamic neurons exhibit patterns of behavioral event-related activity comparable to mPFC? How does the activity of central thalamic nuclei affect action- and outcome-related responses of mPFC neurons?

Early studies revealed similar patterns of elevated firing during memory delays for neurons in MD and dlPFC in monkeys performing delayed response tasks: activity hypothesized to represent information held on-line in working memory (Fuster and Alexander, 1971; Tanibuchi and Goldman-Rakic, 2003; Watanabe and Funahashi, 2004a, b). Delay-related activity in primate MD differs from dlPFC in representing information about motor responses rather than sensory cues. Population vector analyses indicate that MD responses shift from sensory- to motor-related responses during the delay interval, suggesting a role for MD in constructing prospective memory information in dlPFC (Watanabe and Funahashi, 2012). Neurons in adjacent areas of oculomotor thalamus, including the rostral intralaminar nuclei, exhibit visual- and motor saccade-related responses during memory-guided saccade or anti-saccade tasks that resemble responses in reciprocally-connected areas of frontal eye fields in several important ways (Wyder et al., 2003; Tanibuchi and Goldman-Rakic, 2005; Tanaka and Kunimatsu, 2011). Neurons in the oculomotor thalamus differ from frontal eye fields in exhibiting responses more strongly related to movement information and in their sensitivity to differences in cognitive or behavioral demands (Costello et al., 2016). Studies of primate MD and oculomotor thalamus have focused primarily on sensory- and motor-related responses observed in dlPFC and frontal eye fields and not on reward-related responses observed in reciprocally-connected areas of mPFC (Amiez et al., 2006; Matsumoto et al., 2007; Enel et al., 2020).

**Figure 11:**
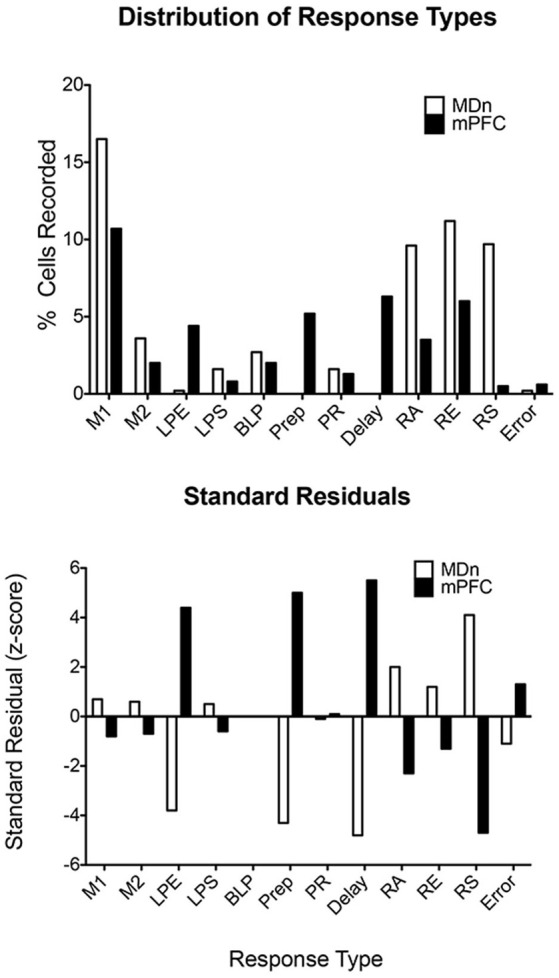
Distribution of response types observed in the mediodorsal thalamic nucleus (MDn) and mPFC during the dDNMTP task. Standard residuals indicate the contributions of each response type to the significant chi-square comparing the distribution of different response types in MDn and mPFC. Results are shown for movement 1 (M1) and 2 (M2), lever press excitation (LPE) and suppression (LPS), base lever press (BLP), preparation (Prep), post-reinforcement (PR), error (E), and reinforcement anticipation (RA), excitation (RE), and suppression (RS). The figure is reproduced from Miller et al. (2017).

The limited evidence available for the rat, suggests that MD neurons represent task-relevant sensory, motor, and reinforcement information (Oyoshi et al., 1996; Han et al., 2013; Courtiol and Wilson, 2016). Miller et al. (2017) compared neuronal responses in MD directly with earlier results for mPFC for rats performing the dDNMTP task. Of 1179 isolated neurons in nine rats, 254 (22%) exhibited criterion event-related responses, 237 (20.1%) with temporal patterns that matched response types in mPFC (Figures 7, 8). The percentage of corresponding responses is consistent with the strong excitatory projections of mPFC to MD. There were disparities in the relative number of different response types (Figure 11): MD had more responses related to movement (45% vs. 29% for mPFC) and reinforcement (51% vs. 27%), relatively few related to lever press actions (2.1% vs. 14.9%), and no responses spanning the memory delay (vs. 12.7% for PFC) or during preparation before the start response (vs. 9.6% for mPFC). The lack of preparatory activity in MD may reflect the role of VM as a thalamic hub for circuits supporting motor preparation (Guo et al., 2017). Choice in dDNMTP is defined by movements towards levers (Figure 6). Choice responses in dDNMTP are associated with increased frequency of movement-related responses and decreased frequency of lever press responses (Francoeur and Mair, 2020). Thus, the preponderance of MD responses related to movement and reward in dDNMTP is indicative of a role in the reward-guided choice. These results are consistent with evidence from studies of monkeys that MD contains numerous neurons that represent information about forthcoming movements during choice responses in ocular delayed response tasks (Watanabe and Funahashi, 2012). The large proportion of MD neurons with reward-related responses during dDNMTP is in keeping with the prominence of reward-related responses in mPFC (Figures 8, 9) and convergent inputs to MD from reward-related areas in the orbitofrontal cortex, ventral pallidum, and amygdala. The lack of delay-related responses in MD is surprising given the prominence of these responses in primate MD during delayed response tasks. This may reflect distinct properties of primate dlPFC (Watanabe and Funahashi, 2012), that lack a homolog in the rodent brain.

**Figure 12:**
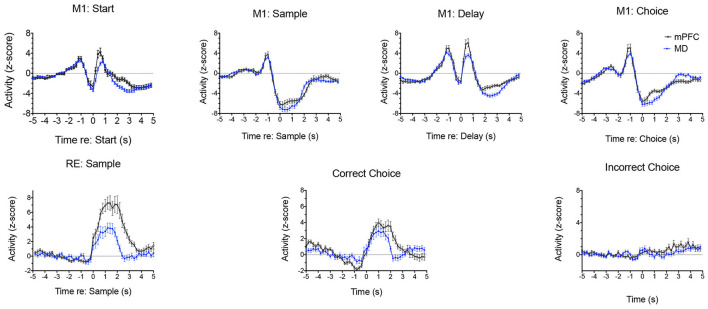
Normalized population PETHs comparing the two most common responses in mediodorsal thalamus (MD) and medial prefrontal cortex (mPFC). Movement 1 (M1) responses aligned with the start, sample, delay, and choice lever presses are based on all samples observed in MD (*n* = 91) and mPFC (*n* = 97). Reinforcement excitation (RE) responses aligned with reinforced sample and correct choice and unreinforced incorrect choice responses are based on all examples observed in MD (*n* = 47) and mPFC (*n* = 63). Error bars represent the standard error of the mean. Data are replotted from Miller et al. (2017).

Analyses of normalized population histograms reveal close correspondence in temporal patterns of activity in MD and mPFC. Figure 12 shows normalized PETHs for the two response types observed most frequently in both MD and mPFC during dDNMTP: movement between all lever presses (M1) and reinforcement excitation (RE). The close timing of these responses seems consistent with the strong excitatory projections from mPFC to MD and the reciprocal thalamocortical projections from MD to mPFC. Reinforcement suppression responses stand out as the one conspicuous MD response type that does not have a corresponding population in mPFC (Figure 11). Ventral pallidum (VP) contains neurons that fire in response to rewards and their predictive stimuli (Ahrens et al., 2016; Ottenheimer et al., 2018; Richard et al., 2018) and provides a robust inhibitory projection to MD that could potentially contribute to these responses (Root et al., 2015). We have recently recorded neuronal activity of VP neurons in rats performing the dDNMTP task and found that 117/177 (68%) of neurons with criterion event-related responses exhibit elevated firing when the reward is delivered, consistent with the timing of reinforcement suppression responses in MD (Krell, 2020).

**Figure 13:**
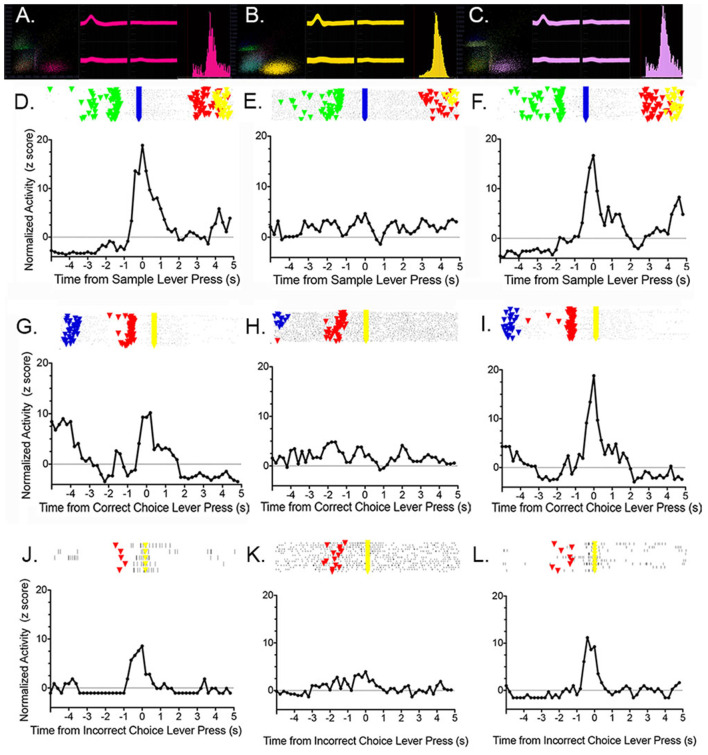
Effects of unilateral thalamic inactivation by 1.0 nmol muscimol near the junction of the paracentral and mediodorsal nuclei on a reinforcement anticipation (RA) response in ipsilateral mPFC. Results are shown for pre-inactivation day 1 **(A,D,G,J)**, inactivation day 2 **(B,E,H,K)**, and post-inactivation day 3 **(C,F,I,L)**. 3D cluster plots, waveforms at each tetrode wire, and the interspike interval histograms **(A,B,C)** confirm the identity of the neuron held across 3 days and show the increase in activity observed during thalamic inactivation. Rasters and normalized PETHs aligned with sample **(D,E,F)**, correct choice **(G,H,I)**, and incorrect choice **(J,K,L)** reveal typical RA responses on days 1 and 3 that disappear during day 2 thalamic inactivation. Figure reproduced from Francoeur et al. (2019).

Francoeur et al. (2019) examined the effects of central thalamic inactivation on mPFC by injecting muscimol at sites (affecting MD and IL) and doses previously found to produce delay independent impairment for DMTP (Figure 4D; Mair and Hembrook, 2008) and sensory-guided choice for VSRT (Newman and Mair, 2007) when applied bilaterally. To avoid disrupting behavior, which is necessary to characterize dDNMTP event-related responses, we inhibited the central thalamus unilaterally and recorded the activity of mPFC neurons in the ipsilateral mPFC. The effects of thalamic inhibition were examined by comparing the activity of single neurons across three sessions, 1 day apart: baseline (no injection), thalamic inhibition (unilateral muscimol injection), and recovery (no injection). Central thalamic inhibition increased the average firing rate for some mPFC neurons and reduced it for others while broadly suppressing event-related responses for actions and outcomes. Figure 13 (from Francoeur et al., 2019) shows results for an mPFC neuron with a reinforcement anticipation response that exhibited increased activity with thalamic inhibition. Figures 13A–C show waveforms recorded at each microwire electrode for all action potentials in each 60 m recording session, with the 3D cluster plots, and inter-spike interval (ISI) histograms recorded. These confirm the identity of the neuron recorded across the 3 days and show the decrease in ISI as activity increased during day 2 inactivation for this neuron. Panels D to L in Figure 13 show raster plots and normalized PETHs on day 1 **(D,G,J)**, day 2 **(E,H,K)**, and day 3 **(F,I,L)** aligned with reinforced sample **(D,E,F)** and correct choice **(G,H,I)** and unreinforced incorrect choice **(J,K,L)**. Event-related responses observed on day 1 largely disappeared with thalamic inhibition on day 2 and recovered substantially on day 3. Averaged across all neurons studied, day 2 thalamic inhibition reduced normalized activity during critical response windows to 46.9% of the day 1 response and this recovered to an average of 79.5% during day 3 recovery. Mixed model ANOVAs revealed significant effects of inactivation on day 2 and significant recovery on day 3 based on normalized activity during critical response windows. These effects did not interact with response type, the effect of thalamic inhibition on average firing rate (increased, decreased, or unchanged), location of neuron in dorsal vs. ventral mPFC, or muscimol dose. These results show that dDNMTP event-related responses are reduced nonspecifically in mPFC with behaviorally-significant inactivation of MD and IL.

Optogenetic studies have provided evidence that MD amplifies and sustains behaviorally-relevant information in PFC (Bolkan et al., 2017; Schmitt et al., 2017; Parnaudeau et al., 2018). These results suggest that MD may help tune mPFC neurons to respond to task-relevant information during adaptive goal-directed behavior. To test this possibility, we compared the effects of unilateral MD lesions made before and after initial dDNMTP training. Neuronal responses were then compared in ipsilateral (experimental) and contralateral (control) mPFC (Francoeur, 2019). The unilateral lesions did not have significant effects on behavioral performance. MD lesions made before training were associated with decreased activity of all mPFC neurons in the lesioned hemisphere and a shift in the predicted direction for event-related responses: namely more lever-press-related and fewer movement-related responses in the lesioned hemisphere. MD lesions made after initial training affected the activity of neurons with criterion event-related responses, but not neurons with uncorrelated activity. Lesions made after initial training did not affect the distribution of response types in mPFC in the lesioned vs. unlesioned hemisphere.

The available results suggest that the central thalamus has important short-term and long-term effects on mPFC function during adaptive goal-directed behavior. In the short term, MD amplifies and sustains neuronal responses in mPFC representing task-relevant information (Bolkan et al., 2017; Schmitt et al., 2017; Parnaudeau et al., 2018). Consistent with this, MD lesions produce delay-dependent impairment of response-related DMTP and DNMTP and other tasks that require flexible responses when action-outcome contingencies change. MD lesions made after initial dDNMTP training affect the activity of mPFC neurons with criterion event-related responses, an effect that could potentially contribute to these behavioral deficits. MD lesions made before initial dDNMTP are associated with fewer movement-related and more lever press-related responses in mPFC of the lesioned than the unlesioned hemisphere. This provides evidence of a longer-term impact of MD on mPFC function and suggests that one function of MD is to tune mPFC neurons to respond to task-specific information important for adaptive responding. This finding seems consistent with evidence that MD lesions made before (but not after) initial training affects the sensitivity of rats to outcome-devaluation, a hallmark of goal-directed action (Balleine, 2019).

Adjacent IL nuclei have more widespread projections than MD, targeting layer 1 of mPFC and related areas of the cerebral cortex and providing the main thalamic input to the striatum. Lesions or inactivation of the IL nuclei, which inevitably affect juxtaposed areas of MD, produce delay independent impairments of DNMTP and DMPT, increase RT for sensory-guided responding in the VSRT task, and can interfere with habitual, rule-based learning. Inactivation of IL and MD has variable effects on mPFC activity, increasing firing of some neurons and reducing firing of others, and has broad effects on the expression of diverse dDNMTP-related responses in mPFC. These results are consistent with the hypothesis that these nuclei regulate information transmission in cortico-cortical and cortico-basal ganglia circuits that give rise to goal-directed behavior (Saalmann, 2014; Perrin and Venance, 2019).

## Conclusions

1. Medial prefrontal cortex (mPFC) supports multiple functions required for adaptive goal-directed behavior: working memory, flexible trial-by-trial response selection, attending to task-relevant information, encoding relationships between actions and their consequences, and organizing and executing action sequences. mPFC lesions produce delay-independent impairments of egocentric (response-related) DMTP and DNMTP tasks that affect RT and accuracy of responding. They spare comparable allocentric tasks.
2. During the dDNMTP task, mPFC functions are served by discrete populations of neurons with responses related to preparation to respond, movements between levers, lever press responses, reinforcement anticipation, delivery of or lack of expected reinforcement, and memory delay following reinforcement. Population analyses show that these different response types effectively tile the temporal interval from when dDNMTP trials are initiated until they end.
3. No individual thalamic nucleus can fully account for the broad effects of mPFC lesions on adaptive goal-directed behavior. Lesions of specific nuclei have distinct effects on behavior consistent with their anatomical connections.
  (a) MD has very limited effects on egocentric DMTP or DNMTP tasks that depend on mPFC. While some reports find no significant effect of MD lesions on these tasks, others have described delay-dependent deficits that spare RT or impaired acquisition that disappears with training. The reports of delay-dependent deficits are consistent with evidence that MD sustains and amplifies neuronal responses that represent behaviorally-relevant information in mPFC. Impairments in the acquisition are consistent with evidence that MD interacts with PFC to detect and encode action-outcome contingencies that are the basis of goal-directed learning.
  (b) Rostral intralaminar and VM nuclei affect speed and accuracy of responding based on learned conditional rules, effects consistent with their prominent connections with striatum and motor cortices, respectively. Like mPFC lesions rostral intralaminar and VM lesions produce delay-independent impairments affecting response speed and accuracy for egocentric DMTP and DNMTP tasks while sparing allocentric DNMTP.
  (c) Anterior thalamic and ventral midline Re and Rh nuclei affect allocentric spatial function, consistent with their prominent connections with the hippocampal system. Anterior thalamic lesions spare egocentric DMTP and DNMTP tasks affected by mPFC lesions. ReRh lesions affect tasks that depend on both mPFC and hippocampus.
  (d) Dorsal midline nuclei integrate inputs from visceral-, arousal-, and emotion-related areas of the brain and influence cortical and subcortical circuits related to mPFC function. They are important for behavioral-state control of adaptive responding and response to salient stimuli in associative learning. Dorsal midline lesions spare DMTP and DNMTP tasks that depend on mPFC.
4. During the dDNMTP task, most MD neurons with criterion event-related responses (237/254) exhibit temporal patterns of firing that closely match response types in mPFC. A preponderance of these are movement and reinforcement-related responses critical for dDNMTP choice. MD lesions made before initial training selectively decrease the number of movement-related responses in mPFC.
5. Drug inactivation of MD and adjacent intralaminar nuclei broadly suppresses the expression of event-related activity in mPFC during the dDNMTP task. Optogenetic studies suggest that MD amplifies and sustains behaviorally-relevant information in the PFC, a process that might help tune mPFC neurons to respond to task-relevant information during goal-directed behavior or suppress the expression of event-related activity during more prolonged drug inactivation.

## Author Contributions

RM was primarily responsible for writing the article. MF and BG contributed to writing and discussion of the manuscript. All authors contributed to the article and approved the submitted version.

## Conflict of Interest

The authors declare that the research was conducted in the absence of any commercial or financial relationships that could be construed as a potential conflict of interest.
